# Adrenomedullin alleviates the pyroptosis of Leydig cells by promoting autophagy via the ROS–AMPK–mTOR axis

**DOI:** 10.1038/s41419-019-1728-5

**Published:** 2019-06-20

**Authors:** Ming-yong Li, Xia-lian Zhu, Bi-xia Zhao, Lei Shi, Wei Wang, Wei Hu, Song-lin Qin, Bing-hai Chen, Pang-hu Zhou, Bo Qiu, Yong Gao, Bo-long Liu

**Affiliations:** 1 Department of Urology, The First Affiliated Hospital of University of South China, No. 69 Chuan Shan Road, Hengyang, 421001 Hunan Province China; 2 Department of Hand Surgery, Affiliated Nanhua Hospital of University of South China, No. 336 Dong Feng South Road, Hengyang, 421002 Hunan Province China; 3 Department of Urology, Affiliated Nanhua Hospital of University of South China, No. 336 Dong Feng South Road, Hengyang, 421002 Hunan Province China; 4 Department of Oncology, Renmin Hospital of Wuhan University, No. 238 Liberation Road, Wuhan, 430060 Hubei Province China; 5 Department of Urology, The First Affiliated Hospital of Anhui Medical University, No. 218 Jixi Road, Hefei, 230022 Anhui Province China; 6 Department of Andrology, The First Affiliated Hospital of University of South China, No. 69 Chuan Shan Road, Hengyang, 421001 Hunan Province China; 7 Department of Urology, Affiliated Hospital of Jiangsu University, No. 438 Liberation Road, Zhenjiang, 212000 Jiangsu Province China; 8 Department of Orthopedics, Renmin Hospital of Wuhan University, No. 238 Liberation Road, Wuhan, 430060 Hubei Province China; 9 Reproductive Medicine Centre, The First Affiliated Hospital of Sun Yat-sen University, No. 58 Second Zhongshan Road, Guangzhou, 510080 Guangdong Province China

**Keywords:** Cell biology, Diseases

## Abstract

Adrenomedullin (ADM) exerts anti-oxidant, anti-inflammatory and anti-apoptotic effects in Leydig cells. However, the role and mechanism of ADM in the pyroptosis of Leydig cells are poorly understood. This study first showed the protective effects of ADM on the pyroptosis and biological functions of Leydig cells exposed to lipopolysaccharide (LPS) by promoting autophagy. Primary rat Leydig cells were treated with various concentrations of LPS and ADM, together with or without N-acetyl-L-cysteine (NAC) or 3-methyladenine (3-MA). Cell proliferation was detected through CCK-8 and BrdU incorporation assays, and ROS level was measured with the DCFDA assay. Real-time PCR, western blot, immunofluorescence, transmission electron microscopy, TUNEL and flow cytometry were performed to examine ADM’s effect on the pyroptosis, autophagy and steroidogenic enzymes of Leydig cells and AMPK/mTOR signalling. Like NAC, ADM dose-dependently reduced LPS-induced cytotoxicity and ROS overproduction. ADM also dose-dependently ameliorated LPS-induced pyroptosis by reversing the increased expression of NLRP3, ASC, caspase-1, IL-1β, IL-18, GSDMD, caspase-3, caspase-7, TUNEL-positive and PI and active caspase-1 double-stained positive rate, DNA fragmentation and LDH concentration, which could be rescued via co-incubation with 3-MA. ADM dose-dependently increased autophagy in LPS-induced Leydig cells, as confirmed by the increased expression of LC3-I/II, Beclin-1 and ATG-5; decreased expression of p62 and autophagosomes formation; and increased LC3-II/LC3-I ratio. However, co-treatment with 3-MA evidently decreased autophagy. Furthermore, ADM dose-dependently rescued the expression of steroidogenic enzymes, including StAR, P450scc, 3β-HSD and CYP17, and testosterone production in LPS-induced Leydig cells. Like rapamycin, ADM dose-dependently enhanced AMPK phosphorylation but reduced mTOR phosphorylation in LPS-induced Leydig cells, which could be rescued via co-incubation with 3-MA. In addition, pyroptosis was further decreased, and autophagy was further promoted in LPS-induced Leydig cells upon co-treatment with ADM and rapamycin. ADM may protect the steroidogenic functions of Leydig cells against pyroptosis by activating autophagy via the ROS–AMPK–mTOR axis.

## Introduction

Leydig cells, located within the interstitial compartment of the testes, mainly contribute to testosterone synthesis and secretion and play a principal role in the development of male traits, reproductive activity and male factor fertility^1^. Bacterial lipopolysaccharide (LPS) can induce oxidative stress that leads to the perturbation of Leydig cell mitochondria, which may be the major influential factor involved in the steroidogenic impairment of Leydig cells^2–4^. Therefore, understanding the cellular and molecular mechanisms underlying the recovery of steroidogenic impairment of Leydig cells has important implications.

The identification of key molecules in the recovery of impaired steroidogenic property of Leydig cells that can be targeted for therapy may help improve outcomes for patients with acute bacterial orchitis. One such potential molecule is adrenomedullin (ADM), which is a possible target for novel therapeutic intervention^5^. ADM is a 52-amino acid peptide originally discovered in human pheochromocytoma tissue and characterised by vasodilation and blood-pressure-lowering effects^6^. Given its anti-oxidant, anti-inflammatory, anti-apoptotic and proliferative properties, ADM exhibits potent protective functions under diverse pathological conditions as an endogenous peptide^7,8^. ADM is present in the human and rat male reproductive systems and plays an important role in modulating the control of testicular steroidogenic functions as a paracrine peptide^9,10^. Our previous studies have demonstrated that ADM causes a restorative effect on steroidogenesis in LPS-treated primary rat Leydig cells by attenuating oxidative stress, inflammation and apoptosis^11,12^.

Cell pyroptosis is a programmed lytic cell death characterised by rapid plasma membrane rupture, DNA damage and release of proinflammatory intracellular contents; however, it is distinct from apoptosis and necrosis in signalling mechanism^13^. This process occurs during infection by many intracellular pathogens, where it can critically eliminate an intracellular replication niche, as well as other settings^14^. Autophagy is a natural, destructive cellular mechanism involving the degradation of cellular components through lysosomal machinery, thus maintaining cellular homeostasis and supplying substrates for energy generation^15^. It is a critical pathway for homeostasis, development and other pathophysiological processes^16^. Autophagy is extremely active in Leydig cells and is involved in steroid production^17^. Cell fate regulation by opposing cell survival and cell death pathways is important for cellular and tissue homeostasis; however, the balance between cell survival and cell death is often lost in human pathologies, such as inflammation^18^. Autophagy plays a critical role in cell survival: essential nutrients are generated via autophagy-dependent degradation and recycling of cellular garbage^19^. Cell death is induced in many cell types by different programs, such as pyroptosis^20^.

To the best of our knowledge, this study is the first to show the autophagy-dependent effect of ADM in testicular Leydig cells. Therefore, we hypothesised that LPS-induced reactive oxygen species (ROS) overproduction could result in pyroptosis of primary rat Leydig cells, consequently impairing the steroidogenic functions of Leydig cells. However, ADM may promote autophagy to alleviate pyroptosis and then rescue the biological functions of Leydig cells via the ROS–AMPK–mTOR axis.

## Results

### ADM reverses the decrease in cell viability and cell proliferation in LPS-exposed Leydig cells

Leydig cells were treated with different concentrations of ADM (0, 10, 50, 100 and 300 nM) for different lengths of time (6, 12 and 18 h). The CCK-8 assay showed that the group treated with 100 nM of ADM for 12 h exerted the strongest effect (Supplementary Fig. 1A). Leydig cells were treated with different concentrations of LPS (0, 0.5, 1.0, 1.5 and 2.0 μg/mL) for 12 h. The CCK-8 assay demonstrated that cell viability decreased in a dose-dependent manner. However, the LPS-induced cytotoxic effect was significantly rescued by the administration of 100 nM of ADM or 10 mM of N-acetyl-L-cysteine (NAC) (Supplementary Fig. 1B). Cell proliferation was also significantly suppressed by 1.0 μg/mL of LPS compared with that in the control group and was significantly reversed by ADM supplementation in a dose-dependent manner or 10 mM of NAC (Supplementary Fig. 1C, D).

### ADM inhibits ROS overproduction in LPS-exposed Leydig cells

Leydig cells were treated with 1.0 μg/mL of LPS with or without ADM or NAC for 12 h. The CCK-8 assay indicated that ROS fluorescence-positive cells were significantly increased compared with that of the control group (Supplementary Fig. 2A). This finding was further confirmed by DCF fluorescence intensity analyses (Supplementary Fig. 2B) and ROS concentration measurements (Supplementary Fig. 2C). However, this phenomenon was ameliorated by ADM administration in a dose-dependent manner or NAC.

### ADM inhibits cell death in LPS-exposed Leydig cells

**Fig. 1:**
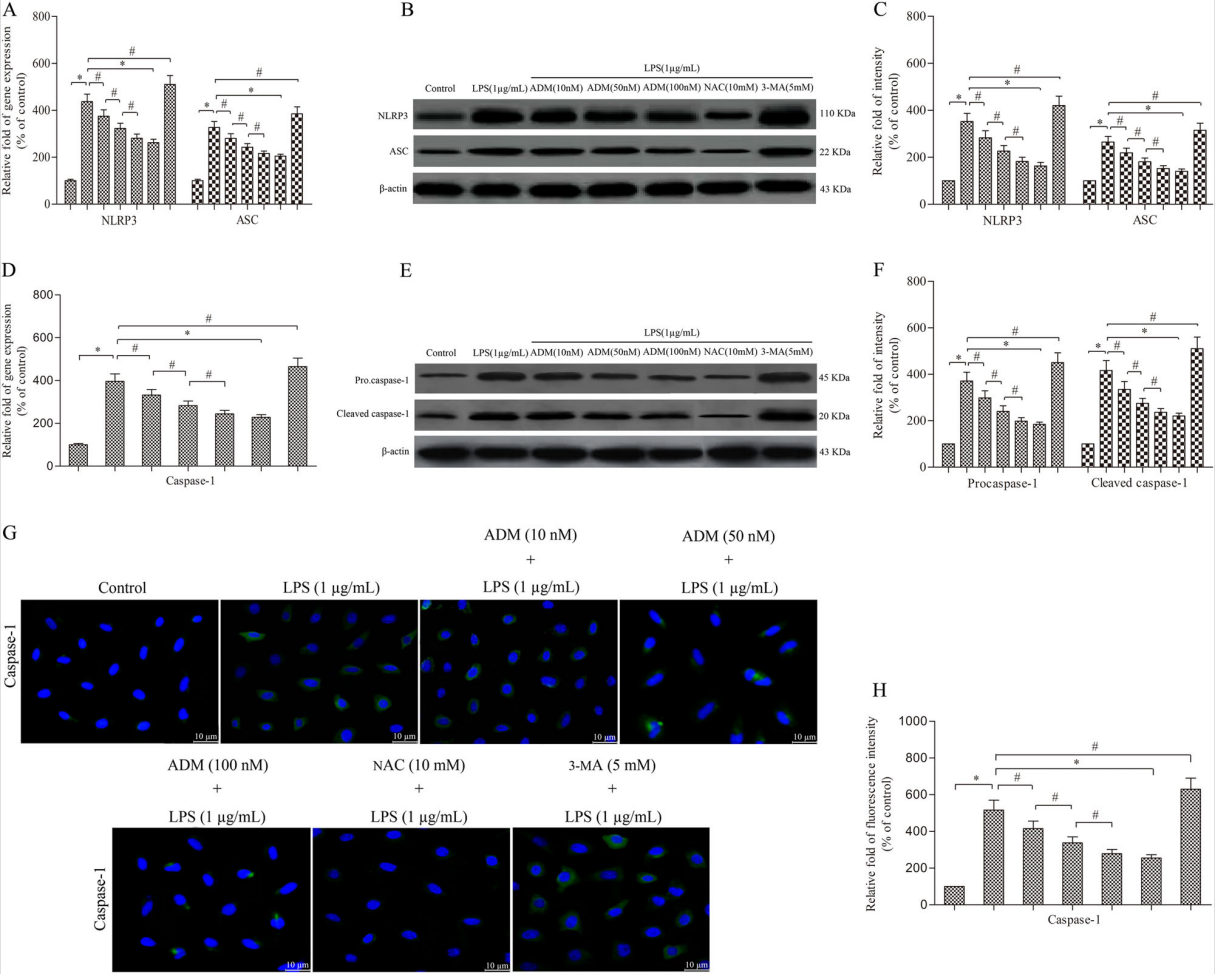
ADM inhibits the expression of NLRP3, ASC and caspase-1 in LPS-exposed Leydig cells. Semi-quantitative real time PCR was performed to detect the gene expression of NLRP3 and ASC (**a**) and caspase-1 (**d**) normalised by the control group. Western blot was used to evaluate the protein level of NLRP3 and ASC (**b**) and caspase-1 (**e**). β-actin was used as internal control. Histogram displaying the densitometric analysis results of NLRP3 and ASC (**c**) and caspase-1 (**f**) normalised by the control group. Immunofluorescence staining was used to detect caspase-1 immunoreactivity (**g**) (scale bar: 10 µm). Fluorescence spectrophotometer was used to analyse the fluorescence intensity (**h**) normalised by the control group. The normalised levels of gene expression are expressed as ratios of the copy number of mRNA and that of β-actin cDNA. Data were obtained from five independent experiments and expressed as mean ± SD. *P < 0.01 and ^#^*P* < 0.05, compared with the corresponding control or treatment group

Leydig cells were treated with 1.0 μg/mL of LPS with or without ADM, NAC or 3-methyladenine (3-MA) for 12 h. Semi-quantitative real-time PCR and western blot showed that LPS stimulation significantly increased the expression of NLRP3, ASC and caspase-1 compared with that of the control group (Fig. 1a–f). Immunofluorescence analyses further confirmed that LPS drastically increased the expression of caspase-1 compared with that of the control group (Fig. 1g, h). However, the LPS-induced effect was significantly reversed by ADM administration in a dose-dependent manner or NAC, but it was aggravated by 3-MA.

**Fig. 2:**
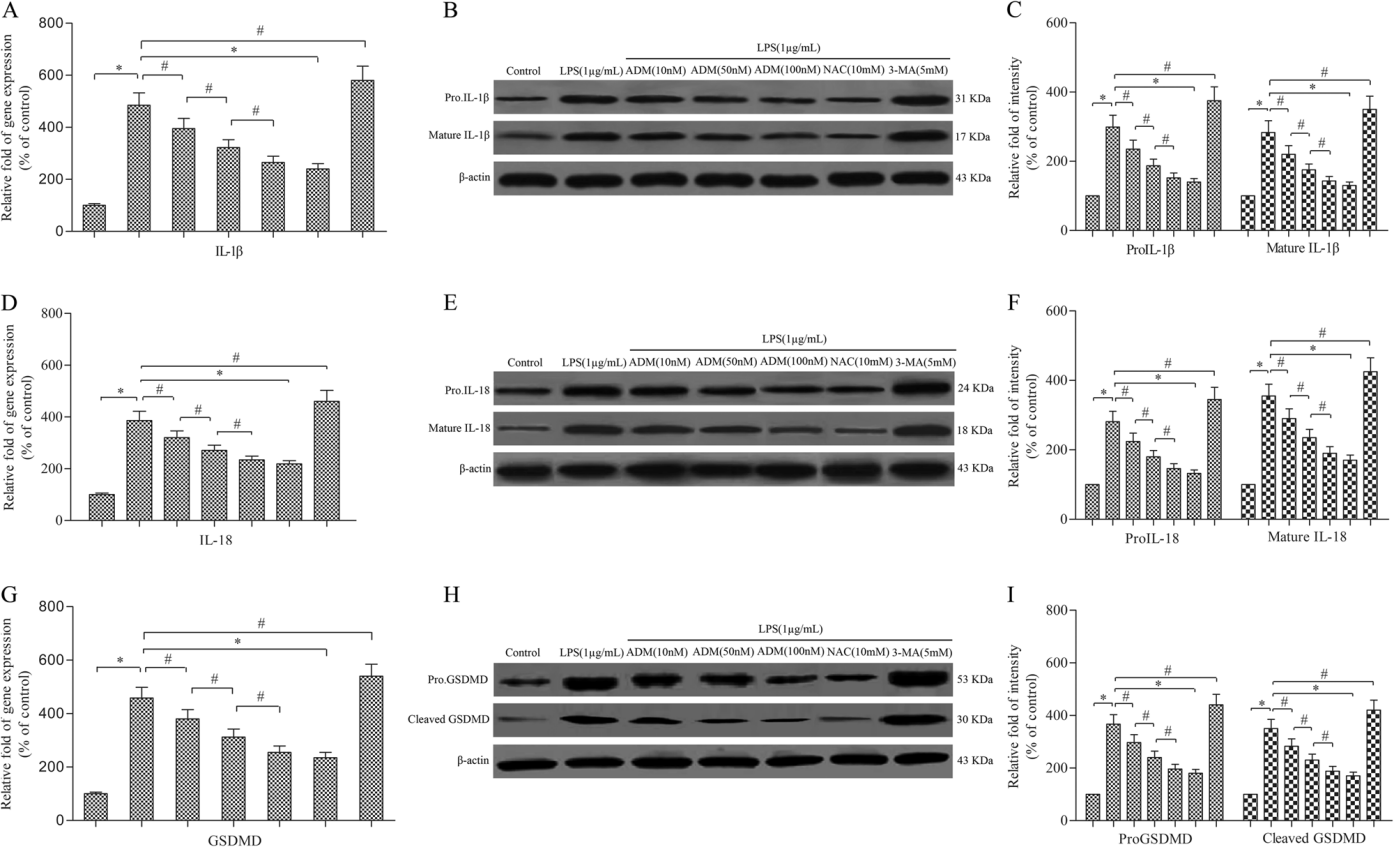
ADM inhibits the expression of IL-1β, IL-18 and GSDMD in LPS-exposed Leydig cells. Semi-quantitative real-time PCR was performed to detect the gene expression of IL-1β (**a**), IL-18 (**d**) and GSDMD (**g**) normalised by the control group. Western blot was used to evaluate the protein level of IL-1β (**b**), IL-18 (**e**) and GSDMD (**h**). β-actin was used as internal control. Histogram displaying the densitometric analysis results of IL-1β (**c**), IL-18 (**f**) and GSDMD (**i**) normalised by the control group. The normalised levels of gene expression are expressed as ratios of the copy number of mRNA and that of β-actin cDNA. Data were obtained from five independent experiments and expressed as mean ± SD. *P < 0.01 and ^#^*P* < 0.05, compared with the corresponding control or treatment group

Semi-quantitative real-time PCR and western blot showed that LPS stimulation significantly increased the expression of IL-1β, IL-18 and GSDMD compared with that of the control group (Fig. 2a–i). However, the expression of IL-1β, IL-18 and GSDMD was rescued by ADM supplementation in a dose-dependent manner or NAC, but it was aggravated by 3-MA. In addition, similar trends were found in the expression of caspase-3 and caspase-7 compared with that of the control group (Supplementary Fig. 3A–F). However, the expression of caspase-3 and caspase-7 was rescued by ADM supplementation in a dose-dependent manner or NAC, but it was aggravated by 3-MA.

**Fig. 3:**
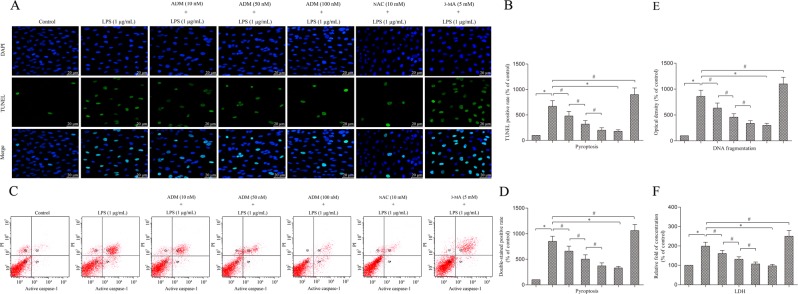
ADM inhibits cell death in LPS-exposed Leydig cells. **a** TUNEL staining was used to evaluate cell death (scale bar: 20 µm). **b** Statistical analysis results of the percentage of TUNEL positive cells normalised by the control group. **c** Flow cytometry was used to evaluate cell death. **d** Statistical analysis results of the percentage of active caspase-1 and PI double-stained positive cells normalised by the control group. **e** Graph displaying DNA fragmentation normalised by the control group. **f** Histogram displaying LDH concentration normalised by the control group. Data were obtained from five independent experiments and expressed as mean ± SD. *P < 0.01 and ^#^*P* < 0.05, compared with the corresponding treatment or control group

Terminal deoxynucleotidyl transferase dUTP nick-end labelling (TUNEL) staining, DNA fragmentation and flow cytometry analyses showed that LPS stimulation significantly increased TUNEL-positive cells, DNA fragmentation and propidium (PI) and active caspase-1 double-stained positive cells compared with that of the control group (Fig. 3a–e). However, the cell death rate was alleviated by ADM addition in a dose-dependent manner or NAC, but it was aggravated by 3-MA. The lactate dehydrogenase (LDH) concentration was also detected to analyse Leydig cell death. The results were consistent with those of TUNEL staining, DNA fragmentation and flow cytometry analyses (Fig. 3f).

### ADM promotes cell autophagy in LPS-exposed Leydig cells

**Fig. 4:**
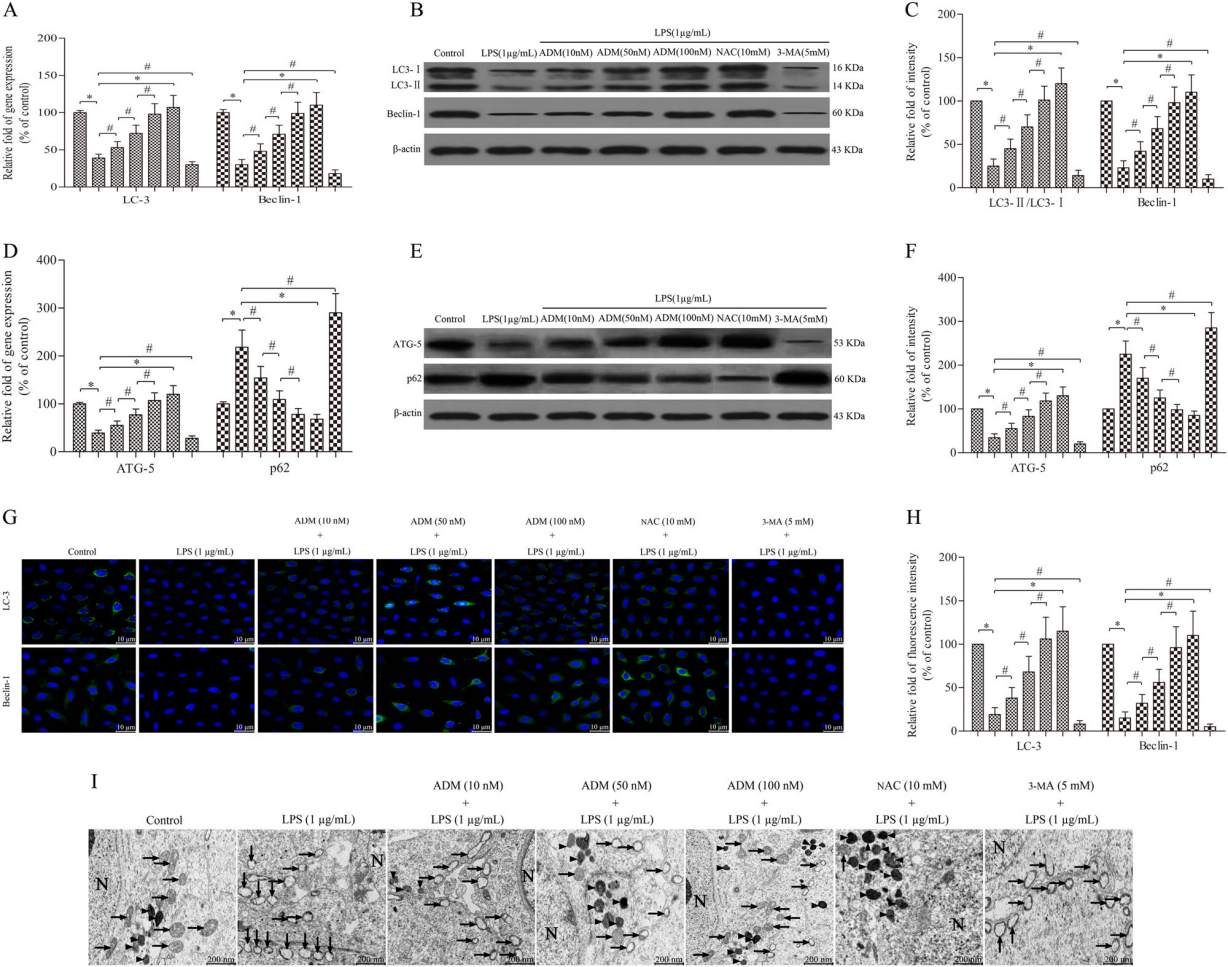
ADM promotes cell autophagy in LPS-exposed Leydig cells. Semi-quantitative real-time PCR was performed to detect the gene expression of LC3 and Beclin-1 (**a**) and ATG-5 and p62 (**d**) normalised by the control group. Western blot was used to evaluate the protein level of LC3 and Beclin-1 (**b**) and ATG-5 and p62 (**e**). β-actin was used as internal control. Histogram displaying the densitometric analysis results of LC3 and Beclin-1 (**c**) and ATG-5 and p62 (**f**) normalised by the control group. Immunofluorescence staining was used to detect LC3 and Beclin-1 immunoreactivity (**g**) (scale bar: 10 µm). Fluorescence spectrophotometer was used to analyse the fluorescence intensity (**h**) normalised by the control group. **i** TEM showed the ultrastructural feature of intracellular mitochondria and autophagosomes (scale bar: 200 nm). Arrow heads show autophagosomes, and arrows show mitochondria. The normalised levels of gene expression are expressed as ratios of the copy number of mRNA and that of β-actin cDNA. Data were obtained from five independent experiments and expressed as mean ± SD. *P < 0.01 and ^#^*P* < 0.05, compared with the corresponding treatment or control group

Leydig cells were treated with 1.0 μg/mL of LPS with or without ADM, NAC or 3-MA for 12 h. Semi-quantitative real-time PCR and western blot showed that LPS stimulation significantly suppressed the expression of LC3 (including LC3-I and LC3-II), Beclin-1 and ATG-5 and increased the expression of p62 compared with that of the control group (Fig. 4a–f). Immunofluorescence analyses further confirmed that LPS drastically increased the expression of LC3 and Beclin-1 compared with that of the control group (Fig. 4g, h). However, the expression of LC3, Beclin-1, ATG-5 and p62 was reversed by ADM supplementation in a dose-dependent manner or NAC, but it was aggravated by 3-MA.

Transmission electron microscopy (TEM) showed that the mitochondria of the cells in the control group were normal with clear mitochondrial cristae, but a few autophagosomes were detected. By contrast, Leydig cells exposed to 1.0 μg/mL of LPS showed intracellular swollen and damaged mitochondria with mitochondrial cristae clouding and evidently decreased number of autophagosomes in the cytoplasm. These effects were reversed by ADM addition in a dose-dependent manner or NAC but were aggravated by 3-MA (Fig. 4i).

### ADM rescues the decrease in steroidogenic enzymes in LPS-exposed Leydig cells

**Fig. 5:**
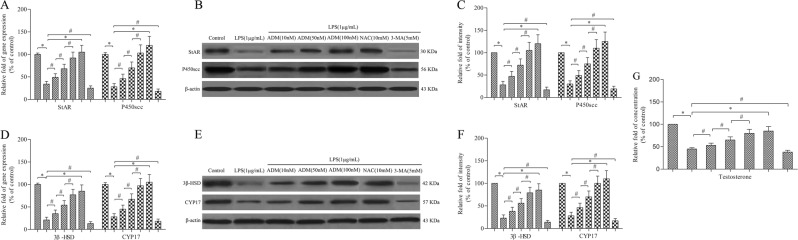
ADM rescues the decrease in steroidogenic enzymes and testosterone production in LPS-exposed Leydig cells. Semi-quantitative real-time PCR was performed to detect the gene expression of StAR and P450scc (**a**) and 3β-HSD and CYP17 (**d**) normalised by the control group. Western blot was used to evaluate the protein level of StAR and P450scc (**b**) and 3β-HSD and CYP17 (**e**). β-actin was used as internal control. Histogram displaying the densitometric analysis results of StAR and P450scc (**c**) and 3β-HSD and CYP17 (**f**) normalised by the control group. **g** Histogram displaying the testosterone concentration normalised by the control group. The normalised levels of gene expression are expressed as ratios of the copy number of mRNA and that of β-actin cDNA. Data were obtained from five independent experiments and expressed as mean ± SD. *P < 0.01 and ^#^*P* < 0.05, compared with the corresponding control or treatment group

Leydig cells were treated with 1.0 μg/mL of LPS with or without ADM, NAC or 3-MA for 12 h. Semi-quantitative real-time PCR and western blot showed that LPS stimulation significantly decreased the expression of StAR, P450scc, 3β-HSD and CYP17 compared with that of the control group (Fig. 5a–f). However, the expression of StAR, P450scc, 3β-HSD and CYP17 was rescued by ADM supplementation in a dose-dependent manner or NAC, but it was aggravated by 3-MA. Testosterone concentration was also detected to analyse the cell biological behaviour of Leydig cells. The trend of results was consistent with those of steroidogenic enzymes (Fig. 3g).

### ADM inhibits the AMPK/mTOR signalling pathway in LPS-exposed Leydig cells

**Fig. 6:**
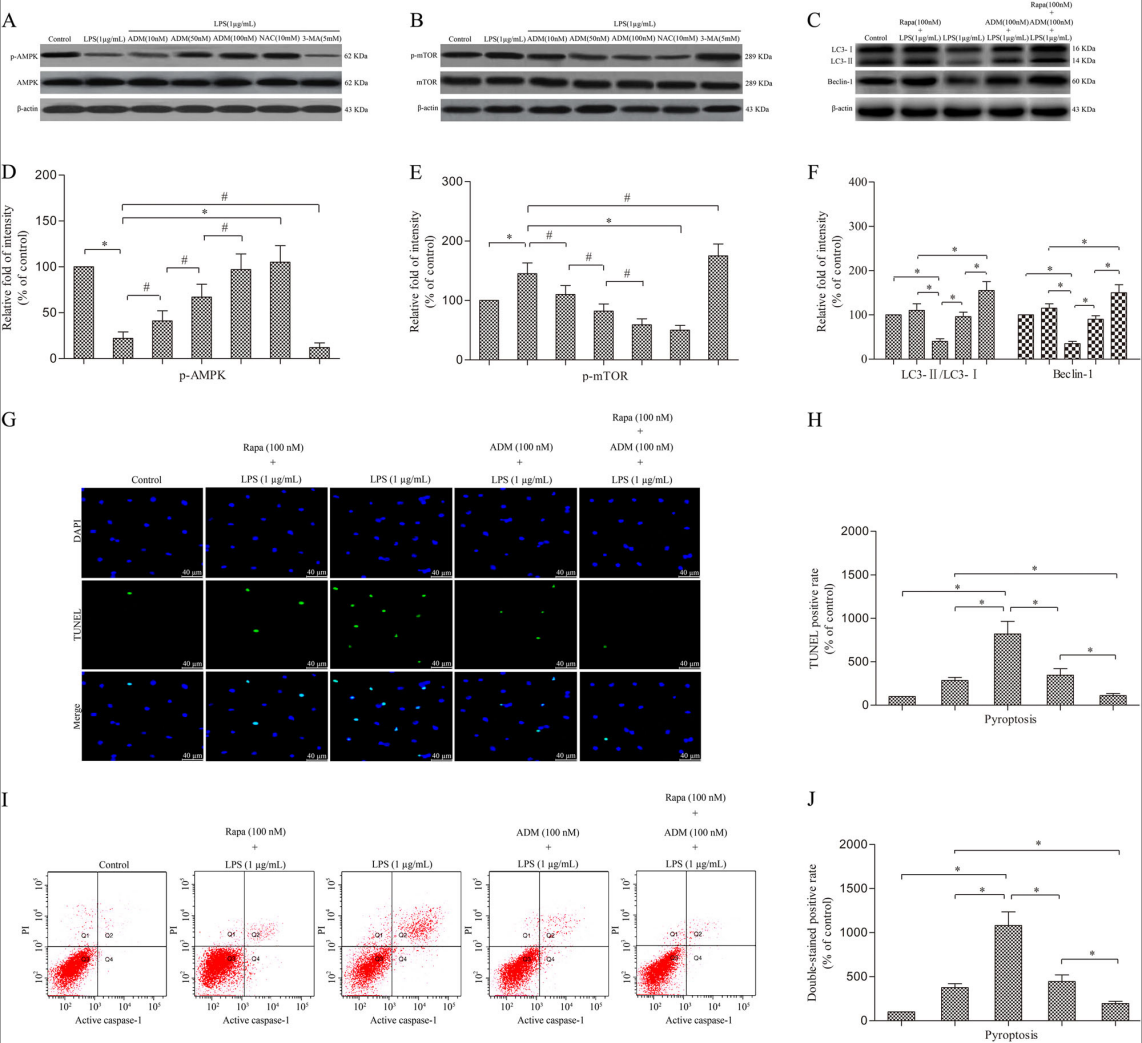
ADM enhances cell autophagy to attenuate pyroptosis by inhibiting the AMPK/mTOR signalling pathway in LPS-exposed Leydig cells. Western blot was used to detect the protein level of p-AMPK and AMPK (**a**) and p-mTOR and mTOR (**b**). Western blot was performed to evaluate the expression of LC3 and Beclin-1 in LPS-induced Leydig cells treated with ADM alone, rapamycin alone or ADM in combination with rapamycin (**c**). β-actin was used as internal control. Histogram displaying the densitometric analysis results of p-AMPK (**d**) and p-mTOR (**e**) and the ratio of LC3-II/LC3-I and Beclin-1 (**f**) normalised by the control group. TUNEL staining was used to evaluate the cell death (**g**) (scale bar: 40 µm). Statistical analysis of the percentage of TUNEL-positive cells normalised by the control group (**h**). **i** Flow cytometry was used to evaluate cell death. **j** Statistical analysis of the percentage of active caspase-1 and PI double-stained positive cells normalised by the control group. The normalised levels of gene expression are expressed as ratios of the copy number of mRNA and that of β-actin cDNA. Data were obtained from five independent experiments and expressed as mean ± SD. *P < 0.01 and ^#^*P* < 0.05, compared with the corresponding control or treatment group

Given that rapamycin induces cell autophagy by inhibiting mTOR expression, we speculate that ADM induces Leydig cell autophagy by inhibiting the AMPK/mTOR signalling pathway similar to rapamycin. After treatment with LPS, western blot showed that the expression of phosphorylated AMPK tended to decrease with the increase of mTOR (Fig. 6a–e). However, the effect of LPS was effectively rescued by ADM in a dose-dependent manner or NAC, but it was aggravated by 3-MA.

### ADM and rapamycin additively enhance cell autophagy to attenuate pyroptosis in LPS-exposed leydig cells

Western blot showed that LPS stimulation significantly suppressed the expression of LC3 (including LC3-I and LC3-II) and Beclin-1 compared with that of the control group. However, ADM and rapamycin evidently enhanced the expression of LC3 (including LC3-I and LC3-II) and Beclin-1 compared with that of the LPS-treated group. Moreover, combined ADM and rapamycin further significantly increased the expression of LC3 (including LC3-I and LC3-II) and Beclin-1 compared with that of the individual groups (Fig. 6c–f).

TUNEL staining and flow cytometry analyses showed that LPS stimulation significantly increased TUNEL-positive and PI and active caspase-1 double-stained positive cells compared with that of the control group. However, ADM and rapamycin evidently reduced the cell death rate compared with that of the LPS-treated group. Furthermore, combined ADM and rapamycin further significantly alleviated the cell death rate compared with that of the individual groups (Fig. 6g–j).

## Discussion

The data gathered in this study suggest that like NAC, ADM dose-dependently alleviated the pyroptosis of Leydig cells exposed to LPS by promoting cell autophagy via the ROS–AMPK–mTOR axis. This effect was confirmed by the additional effect of 3-MA or rapamycin.

The effect of ADM on Leydig cell viability was the strongest when the ADM concentration was 100 nM. ADM could reverse the LPS-induced decrease in Leydig cell viability at the same concentration. ADM could dose-dependently ameliorate the LPS-induced damage of Leydig cells as confirmed by the rescued LPS-induced decrease in Leydig cell proliferation. These findings are consistent with those of our previous reports^8,11,12^. Similarly, the co-incubation of the Leydig cells with NAC effectively reversed the LPS-induced decrease in cell viability and proliferation.

Oxidative stress induced by ROS generation, leading to redox imbalance and lipid peroxidation, is an important mechanism that participates in the cytotoxic effect when it exceeds the capacity of the cell to repair biomolecule oxidation^21^. Our previous study has demonstrated that ADM could attenuate the LPS-induced ROS overproduction via the MAPK/NF-κB signalling pathways^12^. In the current study, the findings further confirmed that like NAC, ADM dose-dependently reduced LPS-induced ROS overproduction, indicating that the decreased ROS generation caused by ADM could be one of the protective mechanisms participating in Leydig cell viability.

ROS, produced by many known activators of NLRP3 inflammasomes, are shown to be a critical mechanism triggering NLRP3 inflammasome formation and activation in response to many exogenous stimuli as well as endogenously produced or secreted molecules from damaged cells^22^. The NLRP3 inflammasome is a multiprotein platform that is activated upon cellular infection or stress and subsequently leads to caspase-1-dependent secretion of proinflammatory cytokines, such as IL-1β and IL-18, and an inflammatory form of cell death termed as pyroptosis^23,24^. The ASC is essential for the best-studied NLRP3 inflammasome to recruit caspase-1 and build an active inflammasome complex^25^. In addition, caspase-1 specifically cleaves the linker between the amino-terminal gasdermin-N and carboxy-terminal gasdermin-C domains in GSDMD, which is required and sufficient for pyroptosis^26^. Our study clarified for the first time that like NAC, ADM could reduce ROS overproduction and mitigate the expression of NLRP3, ASC, caspase-1, IL-1β, IL-18 and GSDMD in a dose-dependent manner, thereby relieving the progress of pyroptosis in LPS-exposed Leydig cells. However, co-incubation with 3-MA aggravated the pyroptosis. Therefore, ADM may be regarded that can suppress pyroptosis resulting from an overwhelming ROS in LPS-exposed Leydig cells. Pyroptosis is a form of programmed cell death resulting in rapid cell lysis that is distinct from apoptosis, which is a non-lytic form of programmed cell death in signalling mechanisms^27^. Caspase activation is the final process of apoptosis, in which caspase-3 and caspase-7 are the most important biomarkers and effectors^28^. Our results showed that like NAC, ADM could relieve apoptosis by rescuing the expression of caspase-3 and caspase-7 in a dose-dependent manner in LPS-exposed Leydig cells, which is consistent with that of our previous report^11^. However, co-incubation with 3-MA aggravated apoptosis. Although the molecular mechanisms of the two types of programmed cell death differ, the cell response to ROS overproduction likely involves multiple programmed cell death pathways, and this condition reveals bidirectional crosstalk between pyroptosis and apoptosis, further illuminating the complex interplay between cell death pathways in LPS-exposed Leydig cells.

Autophagy is also characterised by increased expression levels of autophagy-related proteins, such as LC3-I to LC3-II conversion, Beclin-1 and ATG5, and decreased expression levels of p62^29^. Autophagy can reduce ROS accumulation by clearing damaged mitochondria, which are the main sources of free radicals^30^. The primary source of the excessive production of ROS is the mitochondria with the capacity to exceed the production of endogenous anti-oxidants^31^. Autophagy and apoptosis occur when cells are under stress, and autophagy usually precedes apoptosis and maintains cell homeostasis^32^. Our previous study has shown that ADM could protect rat Leydig cells from LPS-induced inflammation and apoptosis via the PI3K/Akt mitochondrial signalling pathway^11^. Our present results showed that like NAC, ADM increased the expression of LC3, Beclin-1 and ATG-5 but decreased that of p62 in a dose-dependent manner in LPS-exposed Leydig cells, thereby promoting the progression of cell autophagy. However, co-incubation with 3-MA decreased the autophagy process. Since autophagy was discovered, it has been characterised by autophagosome formation and thought to act as a pro-survival response to several stresses by providing recycled metabolic substrates to maintain energy homeostasis^33^. In our study, like NAC, ADM reduced the number of intracellular swollen and damaged mitochondria with mitochondrial cristae clouding and increased the number of double-membrane structures resembling autophagosomes in a dose-dependent manner in LPS-exposed Leydig cells. However, co-incubation with 3-MA decreased autophagosome formation. Autophagy modulation could protect against multiple organ injuries by inhibiting the NLRP3 inflammasome and ameliorating NLRP3-associated complications^34^. Impaired autophagy may contribute to the aberrant activation of some signalling pathways, leading to uncontrolled inflammation and cell death^35^. Considering these results, we speculate that ADM could alleviate Leydig cell pyroptosis resulting from LPS-induced overwhelming ROS by promoting autophagy to clear excessive ROS and damaged mitochondria.

Testicular testosterone synthesis occurs in the mitochondria and is regulated by the mitochondria-targeted StAR protein, which controls cholesterol delivery into the mitochondrial matrix, a rate-limiting step in steroid hormone formation^36^. Cholesterol delivered into the mitochondrial matrix is further converted to pregnenolone via cytochrome P450scc and sequentially converted to testosterone by the step-by-step action of 3β-HSD and CYP17^37^. Autophagy promotes cholesterol uptake into Leydig cells, and autophagy dysfunction might inhibit testosterone production^38^. Autophagic deficiency is related to steroidogenic decline in Leydig cells, which might be influenced by intracellular ROS^39^. In the present study, like NAC, ADM dose-dependently improved the expression of StAR, P450scc, 3β-HSD and CYP17 in LPS-exposed Leydig cells, thereby improving testosterone production. However, co-incubation with 3-MA aggravated the expression of steroidogenic enzymes and testosterone synthesis. Our previous study has shown that ADM could reverse the LPS-induced inhibition of testosterone synthesis by reducing the excessive production of ROS^11^. Taken together, ADM could improve testosterone synthesis in LPS-exposed Leydig cells by rescuing the inhibited activity of some steroidogenic enzymes, indicating its therapeutic potential in LPS-induced Leydig cell dysfunction.

We further investigated whether the AMPK/mTOR signalling pathway is involved in the autophagy activation of ADM in LPS-exposed Leydig cells. Several studies have reported that the regulation of autophagy is related to the AMPK/mTOR signalling pathway^40–42^. As a sensor of energy molecules, AMPK is a positive regulator of autophagy and acts by down-regulating mTOR phosphorylation to adapt to energy metabolism^43^. Our results suggest that like NAC, ADM could reduce LPS activity, an effect that promoted AMPK phosphorylation and attenuated mTOR phosphorylation, subsequently leading to autophagy activation. However, co-incubation with 3-MA increased the effect of LPS. This effect verified that the AMPK/mTOR signalling pathway can activate autophagy. ADM may exert its protective effect on LPS-exposed Leydig cells by activating autophagy via the AMPK/mTOR signalling pathway. Rapamycin, which acts through specific inhibition of mTOR phosphorylation in the AMPK/mTOR pathway, is recognised as a drug that promotes autophagy. ADM and rapamycin reduced mTOR phosphorylation; thus, when the two were combined, any increase in autophagy should be more significant. When rapamycin was added together with ADM treatment, the enhancement of autophagy and the decrease of pyroptosis were more significant. These results indicate that the induction of autophagy by ADM may be mediated by the inhibition of the AMPK/mTOR signalling pathway. Further functional experimental results confirmed our hypothesis.

The emerging role of ADM in LPS-exposed Leydig cells may open a new therapeutic approach. However, the precise mechanism of ADM remains to be elucidated, and these findings remains to be established in in vivo conditions. In summary, this is the first study to demonstrate that ADM may protect Leydig cells from pyroptosis caused by LPS-induced overwhelming ROS via activating autophagy, and its effect is mediated through the ROS–AMPK–mTOR axis.

## Materials and methods

### Reagents

Rat ADM (1–50) was obtained from Phoenix (Belmont, CA, USA). LPS from *Escherichia coli*, serotype (O127:B8) was from Sigma (St. Louis, MO, USA). Dulbecco's modified Eagle's medium with Ham's F-12 nutrient mixture (DMEM-F12, at a 1:1 ratio), collagenase type IV, Percoll, trypan blue, bovine serum albumin, foetal bovine serum, 3,3′-diaminobenzidine, 6-diamidino-2-phenylindole dihydrochloride (DAPI), PI, penicillin/streptomycin, NAC and 3-MA were from Gibco (Grand Island, NY, USA). Cell counting kit-8 (CCK-8) assay kits were from Dojindo Laboratories (Kyushu, Japan). The 5-bromo-2′-deoxyuridine (BrdU) cell proliferation assay kits were from Millipore (Billerica, MA, USA). The 2′,7′-dichlorofluorescin diacetate (DCFDA)-cellular ROS detection assay kits were from Abcam (Cambridge, MA, USA). In Situ Cell Apoptosis and LDH Detection kits were from Roche Diagnostics (East Sussex, UK). FLICA 660 in vitro active caspase-1 detection kit was from ImmunoChemistry Technologies (Bloomington, MN, USA). Testosterone enzyme immunoassay (EIA) kit was purchased from Diagnostic Systems Laboratories (Webster, TX, USA). Cell death detection enzyme-linked immunosorbent assay (ELISA) kits were from R&D Systems (Minneapolis, MN, USA). The bicinchoninic acid protein assay kits were from Thermo Fisher Scientific (Waltham, MA, USA). DNeasy tissue extraction kits and RNeasy Plus Mini RNA extraction kits were from Qiagen (Valencia, CA, USA). TaqMan gene expression assays and real-time PCR master mix were from Applied Biosystems (Foster City, CA, USA). Rabbit monoclonal antibodies against LC3-I/II (Cat. No.: 12741S), StAR (Cat. No.: 8449S), P450scc (Cat. No.: 14217S), AMPK (Cat. No.: 5831S), p-AMPK (Cat. No.: 2537S), mTOR (Cat. No.: 2983S) and p-mTOR (Cat. No.: 5536S) were from Cell Signalling Technology (Beverly, MA, USA) and rabbit monoclonal antibody against GSDMD (Cat. No.: ab219800) was from Abcam (Cambridge, England, UK). Rabbit polyclonal antibody against NLRP3 (Cat. No.: bs-10021R) was from Bioss (Edinburgh, Scotland, UK). Mouse monoclonal antibodies against ASC (Cat. No.: sc-514414), caspase-1 (Cat. No.: sc-398715), caspase-3 (Cat. No.: sc-271759), caspase-7 (Cat. No.: sc-365034), IL-1β (Cat. No.: sc-52012), Beclin-1 (Cat. No.: sc-48341), ATG5 (Cat. No.: sc-133158), p62 (Cat. No.: sc-48373), 3β-HSD (Cat. No.: sc-515120) and β-actin (Cat. No.: sc-517582), goat polyclonal antibody against IL-18 (Cat. No.: sc-6179) and rabbit polyclonal antibody against CYP17 (Cat. No.: sc-66850) were from Santa Cruz Biotechnology (Santa Cruz, CA, USA). The FITC- and HRP-conjugated IgG antibodies were from Molecular Probes (Eugene, OR, USA) and Cell Signalling Technology (Beverly, MA, USA), respectively. All other chemicals used in this study were of analytical grade and obtained from Sigma (St. Louis, MO, USA) unless otherwise stated.

### Leydig cell isolation, purification and identification

Leydig cells were isolated from the testes of Sprague–Dawley rats aged ~90 days old in accordance with our previous method^11,12^. Briefly, the testes were decapsulated and digested with DMEM-F12 containing 0.25 mg/mL collagenase. Subsequently, the cell supernatant containing Leydig cells were filtered through a 100 µm nylon mesh (Spectrum, Rancho Dominguez, California). The cells were collected via centrifugation and purified by using discontinuous Percoll gradients (5, 30, 58 and 70%). These purified Leydig cells were carefully collected using a Pasteur pipette and transferred into centrifuge tubes containing DMEM-F12.

Leydig cell viability was estimated by measuring the percentage of cells that excluded trypan blue staining as previously described^14,15^. Briefly, isolated Leydig cells were incubated with an equal volume of 0.4% trypan blue and then an aliquot of cells was loaded into a haemocytometer chamber for counting the number of nonviable (stained) and viable (excluded) cells. Viability was calculated as the percentage of viable cells divided by the total cell count. Leydig cells with at least 95% viability were used for the subsequent experiments.

Leydig cell purity was assessed using histochemical staining for 3β-HSD activity as previously described^44^. Briefly, purified Leydig cells were incubated in phosphate buffer solution (PBS) containing 0.2 mg/mL of nitroblue tetrazolium, 1 mg/mL of nicotinamide adenine dinucleotide and 0.12 mg/mL of dehydroepiandrosterone for 90 min at 34 °C. The percentage of positively stained cells with distinct blue reaction product was counted under an inverted microscope (Olympus, Tokyo, Japan). Leydig cells showed intense staining and were 90% enriched.

### Cell culture and experimental design

Leydig cells were cultured with DMEM-F12 containing 3% FBS at 37 °C for 24 h under 5% CO_2_ and 95% air. At the end of incubation, the cells were incubated with serum-free medium for 1 h, and the culture reached 80% confluence prior to the onset of experimental treatments.

Cells were cultured in 96-well plates with 200 µL of DMEM-F12 containing 3% FBS in the presence of various doses of ADM (0, 10, 50, 100 and 300 nM) at various time points (6, 12 and 18 h) to determine the dose- and time-dependent effects of ADM. Cell viability was measured via the CCK-8 assay.

Cells were incubated in 96-well plates with 200 µL of DMEM-F12 containing 3% FBS supplemented with 100 nM of ADM or 10 mM of NAC for 2 h before various doses of LPS (0.5, 1.0, 1.5 and 2.0 µg/mL) were added. The CCK-8 assay was performed to detect cell viability 12 h after incubation.

For other experiments, cells were cultured in 6-well plates with 2 mL of DMEM-F12 containing 3% FBS or 24-well plates with 1 mL of DMEM-F12 containing 3% FBS. The cells were then divided into the following groups: control (cells were cultured in DMEM-F12 containing 3% FBS only), LPS (cells were cultured in DMEM-F12 containing 3% FBS supplemented with 1 µg/mL of LPS for 12 h), LPS + 10 nM ADM (cells were cultured in DMEM-F12 containing 3% FBS supplemented with 10 nM of ADM for 2 h followed by 12 h with 1 µg/mL of LPS), LPS + 50 nM ADM (cells were cultured in DMEM-F12 containing 3% FBS supplemented with 50 nM of ADM for 2 h followed by 12 h with 1 µg/mL of LPS), LPS + 100 nM ADM (cells were cultured in DMEM-F12 containing 3% FBS supplemented with 100 nM of ADM for 2 h followed by 12 h with 1 µg/mL of LPS), LPS + 10 mM NAC (cells were cultured in DMEM-F12 containing 3% FBS supplemented with 10 mM of NAC for 2 h followed by 12 h with 1 µg/mL of LPS), LPS + 5 mM 3-MA (cells were cultured in DMEM-F12 containing 3% FBS supplemented with 5 mM of 3-MA for 2 h followed by 12 h with 1 µg/mL of LPS), LPS + 100 nM rapamycin (cells were cultured in DMEM-F12 containing 3% FBS supplemented with 100 nM of rapamycin for 2 h followed by 12 h with 1 µg/mL of LPS) and LPS + 100 nM rapamycin + 100 nM ADM (cells were cultured in DMEM-F12 containing 3% FBS supplemented with 100 nM of rapamycin and 100 nM of ADM for 2 h followed by 12 h with 1 µg/mL of LPS).

### CCK-8 assay

After implementing the above-described experimental design and the Leydig cells reached confluence, the growth medium was removed and the wells were washed thrice with 0.1 M of PBS. Cell viability was measured using the CCK-8 assay kits in accordance with the manufacturer's instructions. The absorbance at 450 nm was measured using a microplate reader (PerkinElmer, Waltham, MA, USA).

### BrdU assay

After implementing the above-described experimental design and the Leydig cells reached confluence, the growth medium was removed and the wells were washed thrice with 0.1 M of PBS. Cell proliferation was measured using the BrdU cell proliferation kits in accordance with the manufacturer's instructions. The cells were stained with DAPI for nuclear counterstaining and immunofluorescent photographs were captured under an inverted microscope (Olympus, Tokyo, Japan). Cell counts were performed on randomly selected areas of the BrdU-stained Leydig cells (visual field at ×200 magnification). We counted the number of BrdU-positive cells against the total cell number to calculate the proliferation index (BrdU^+^ cells/total cells).

### ROS measurement

After Leydig cells were treated in accordance with the above-described experimental design and reached confluence, cellular ROS production was measured using a DCFDA assay kit in accordance with the manufacturer's instructions. The cells were subjected to fluorospectrophotometric analysis and fluorescence microscopy in a Wallac 1420 microplate reader (PerkinElmer, Waltham, MA, USA) at an excitation wavelength of 488 nm and emission wavelength of 520 nm. The amount of intracellular ROS was proportional to DCF fluorescence intensity, which was recorded directly to indicate the relative amount of ROS. Relative changes in DCF fluorescence were expressed as fold increase over the control cells.

### Immunofluorescent staining

After Leydig cells were treated in accordance with the above-described experimental design and reached confluence, the cells were fixed with 4% paraformaldehyde at 4 °C for 15 min and permeabilised with 0.2% Triton X-100 in PBS at room temperature for 15 min. Then, the cells were incubated with primary antibodies against caspase-1 (1:200), LC3-I/II (1:200) and Beclin-1(1:200) at 4 °C overnight. Subsequently, 100 μL/well working solution of appropriate secondary antibodies (1:300) was added and incubated at room temperature for 90 min. After the cells were washed with PBS, they were stained with DAPI for nuclear counterstaining. The stained slides were photographed using an inverted microscope (Olympus, Tokyo, Japan). Relative changes of LC3-I/II and Beclin-1 fluorescence were expressed as fold increase over the control cells.

### TUNEL staining

After Leydig cells were treated in accordance with the above-described experimental design and reached confluence, cell death was assessed using a TUNEL assay kit in accordance with the manufacturer's protocol. The cells were stained with DAPI for nuclear counterstaining and observed under an inverted microscope (Olympus, Tokyo, Japan). Images were randomly selected from two sections of each specimen. The fold increase in cell death rate was normalised to that of the control group.

### Flow cytometry

After Leydig cells were treated in accordance with the above-described experimental design and reached confluence, cell death was assessed with FLICA 660-YVADFMK in accordance with the manufacturer's protocol. The cells were stained with PI to mark cells with membrane pores and analysed via flow cytometry (BD Biosciences, San Jose, CA, USA). The fold increase in cell death rate was normalised to that of the control group.

### TEM examination

After Leydig cells were treated in accordance with the above-described experimental design and reached confluence, TEM was used to observe autophagosomes in Leydig cells. After the cells were washed with PBS and harvested with a plastic cell scraper, they were pelleted via centrifugation at 800 rpm for 10 min and fixed in ice-cold 2.5% glutaraldehyde for 2 h. Subsequently, samples were post-fixed in 1% OsO_4_ for 1 h, dehydrated through an ethanol series and embedded in epoxy resin. Finally, the ultrathin sections (60 nm) were double stained with uranyl acetate and lead citrate and examined via TEM (Hitachi 800, Tokyo, Japan).

### DNA fragmentation

After Leydig cells were treated in accordance with the above-described experimental design and reached confluence, intracellular DNA fragmentation in the cell lysates was detected using a cell death detection ELISA kit in accordance with the manufacturer's protocol. Optical density was spectrophotometrically determined at 405 nm with a Wallac 1420 microplate reader (PerkinElmer, Waltham, MA, USA). The fold increase in DNA fragmentation was normalised to that of the control group.

### LDH release

After Leydig cells were treated in accordance with the above-described experimental design and reached confluence, we measured LDH release using an LDH assay kit. Absorbance was measured with a Wallac 1420 microplate reader (PerkinElmer, Waltham, MA, USA) at a wavelength of 490 nm. Background optical absorbance was measured at 690 nm and was subtracted from primary measurements for each well. LDH content in the medium was calculated using a concurrent standard curve. The fold increase in LDH concentrations was normalised against the control group.

### Testosterone measurement

Medium concentrations of testosterone were measured using a testosterone EIA kit in accordance with the manufacturer's protocol. The absorbance at 450 nm was read with a Wallac 1420 microplate reader (PerkinElmer, Waltham, MA, USA). A concurrent standard curve was used to determine testosterone in the culture supernatant. Testosterone concentrations were expressed as ng/mL and normalised against the control group.

### Semi-quantitative real-time PCR

After Leydig cells were treated in accordance with the above-described experimental design and reached confluence, the treated cells were dissolved in Trizol reagent. Total RNA was extracted with the RNeasy Plus Mini RNA extraction kit in accordance with the manufacturer's instructions. RNA concentration was determined using a spectrophotometer (Evolution 220, Waltham, MA, USA) at 260 nm wavelength. Purity was assessed by measuring the ratio of A260/A280, and purified RNA between 1.8 and 2.0 was used in this study. The first strand complimentary DNA was synthesised from RNA via reverse transcriptase and a PrimeScript RT reagent kit (Fermentas, Waltham, MA, USA). Semi-quantitative real-time PCR was performed in a 7900HT Fast Real-Time PCR system (Applied Biosystems, Foster City, CA, USA). Each reaction was run in triplicate and performed in 40 cycles consisting of the following steps: initial denaturation at 95 °C for 5 min followed by a set cycle of denaturation at 94 °C for 10 s and different annealing temperatures for each pair of primers (ranging between 53 and 62 °C) for 10 s, extension at 72 °C for 28 s and a final elongation at 72 °C for 5 min. As a final step, a melt curve analysis was performed. Primer sequences of the targeted genes used in this study were as follows: NLRP3 (5′-CCTGGGGGACTTTGGAATCAG-3′, forward; 5′-GATCCTG ACAACACGCGGA-3′, reverse), ASC (5′-GTGGGTGGCTTTCCTTGATT-3′, forward; 5′-TTGTCTT GGCTGGTGGTCTCT-3′, reverse), caspase-1 (5′-TTTCCGCAAGGTTCGATTTTCA-3′, forward; 5′- GGCATCTGCGCTCTACCATC-3′, reverse), caspase-3 (5′-CGATTATGCAGCAGCCTCAA-3′, forward; 5′-AGGAGATGCCACCTCTCCTT-3′, reverse), caspase-7 (5′-CTACCGCCGTGGGAACGA TG-3′, forward; 5′-CGAAGGCCCATACCTGTCAC-3′, reverse), IL-1β (5′-GCCCATCCTCTGTGAC TCAT-3′, forward; 5′-AGGCCACAGGTATTTTGTC-3′, reverse), IL-18 (5′-GCCTGTGTTCGAGGA TATGACT-3′, forward; 5′-CCTTCACAGAGAGGGTCACAG-3′, reverse), GSDMD (5′-CCAGCATG GAAGCCTTAGA G-3′, forward; 5′-CAGAGTCGAGCACCAGACAC-3′, reverse), LC3 (5′-GAGTG GAAGATGTCCGG CTC-3′, forward; 5′-CCAGGAGGAAGAAGGCTTGG-3′, reverse), Beclin-1 (5′-AGCACGCCATGTATAGCAAAGA-3′, forward; 5′-GGAAGAGGGAAAGGACAGCAT-3′, reverse), ATG-5 (5′-TGAAGGAAGTTGTCTGGATAGCTCA-3′, forward; 5′-AAGTCTGTCCTTCC GCAGTC-3′, reverse), p62 (5′-AAGCCGGGTGGGAATGTTG-3′, forward; 5′-GCTTGGCCCTTCGG ATTCT-3′, reverse), StAR (5′-GCAGCAGGCAACCTGGTG-3′, forward; 5′-TGATTGTCTTCGGCA GCC-3′, reverse), P450scc (5′-GGAGGAGATCGTGGACCCTGA-3′, forward; 5′-TGGAGGCATGTT GAGCATGG-3′, reverse), 3β-HSD (5′-AGCAAAAAGATGGC CGAGAA-3′, forward; 5′-GGCACAA GTATGCAATGTGCC-3′, reverse), CYP17 (5′-ACTGAGGGT ATCGTGGATGC-3′, forward; 5′-TC GAACTTCTCCCTGCACTT-3′, reverse) and β-actin (5′-CGTTG ACATCCGTAAAGAC-3′, forward; 5′-TGGAAGGTGGACAG TGAG-3′, reverse). Specific PCR products subjected to melting curve analysis for each primer set revealed only one peak for each product. All the gene expression levels were normalised for expression of the housekeeping gene, β-actin, and expressed as the fold ratio compared with the control group.

### Western blot

After Leydig cells were treated in accordance with the above-described experimental design and reached confluence, the entire cell lysates were harvested from Leydig cell monolayers. Total protein was adjusted to equal amounts, and protein mixtures were separated via SDS-PAGE and transferred to polyvinylidene difluoride membranes. After the transfer, non-specific binding sites of the membranes were blocked for 1 h at room temperature in PBS (pH 7.4) containing 5% (wt/vol) non-fat dry milk and then incubated with primary antibodies against NLRP3 (1:1000), ASC (1:1000), caspase-1 (1:200), caspase-3 (1:200), caspase-7 (1:200), IL-1β (1:400), IL-18 (1:400), GSDMD (1:1000), LC3-I/II (1:1000), Beclin-1 (1:1000), ATG5 (1:500), p62 (1:500), StAR (1:1000), P450scc (1:1000), 3β-HSD (1:500), CYP17 (1:500), p-AMPK (1:1000), AMPK (1:1000), p-mTOR (1:1000) and mTOR (1:1000) at 4 °C overnight. The membranes were probed with an anti-β-actin antibody (1:1000) to control protein loading, and then incubated for 2 h at room temperature with HRP-conjugated secondary antibodies (1:1000). The results were scanned using a gel imaging system (UVP Company, Upland, CA, USA). Densitometry measurements were performed with Image Lab software (Bio-Rad Laboratories, Hercules, CA, USA). The band intensities were semi-quantified via densitometry analysis using Quantity-One software (Bio-Rad Laboratories, Hercules, CA, USA). Relative protein expression was normalised to β-actin and compared with the control group.

### Statistical analysis

Data were expressed as mean ± standard deviation on the basis of at least five separate experiments. Data were analysed using SPSS version 19.0 (SPSS Inc., Chicago, IL, USA). Significant differences amongst the mean values of multiple groups were evaluated with one-way ANOVA followed by Student–Newman–Keuls’ method. A two-sided *P* value < 0.05 was considered statistically significant.

### Ethical approval

This study followed the national guidelines and protocols of the National Institutes of Health and was approved by the Local Ethics Committee for the Care and Use of Laboratory Animals of the University of South China.

## Supplementary information

Supplementary Figure legends

Supplementary Figure 1

Supplementary Figure 2

Supplementary Figure 3
