# Supplementary material for: Adrenomedullin alleviates the pyroptosis of Leydig cells by promoting autophagy via the ROS–AMPK–mTOR axis
Source: Cell Death Dis. 2019 Jun 20;10(7):489. doi: 10.1038/s41419-019-1728-5 (PMC6586845; doi:10.1038/s41419-019-1728-5)
Supplement: Supplementary file 1 — Supplementary Figure legends [file 41419_2019_1728_MOESM1_ESM.docx]

Supplementary Figure 1. ADM reverses the decrease in cell viability and cell proliferation in LPS-exposed Leydig cells. (A) CCK-8 assay was used to verify the viability of Leydig cells treated with different concentrations of ADM (0, 10, 50, 100 and 300 nM) for 6, 12 and 18 h. (B) CCK-8 assay was used to assess the viability of Leydig cells pretreated with 100 nM of ADM or 10 mM of NAC for 2 h and incubated with different concentrations (0, 0.5, 1.0, 1.5 and 2.0 µg/mL) of LPS for 12 h. (C) BrdU assay was used to detect the proliferation of Leydig cells pretreated with different concentrations of ADM (10, 50 and 100 nM) or 10 mM of NAC for 2 h and incubated with 1.0 µg/mL of LPS for 12 h (scale bar: 20 µm). (D) Statistical analysis results of the percentage of BrdU-positive cells normalised by the control group. Data were obtained from five independent experiments and expressed as mean ± SD. ^*^*P* < 0.01,^**^*P* < 0.05, ^#^*P* < 0.05, compared with the corresponding treatment or control group.

Supplementary Figure 2. ADM inhibits ROS overproduction in LPS-exposed Leydig cells. (A) DCFDA assay was used to evaluate the positive cells of ROS-DCF fluorescence in different treatment groups. The phase images showing equal cell density in the six groups (scale bar: 20 µm). (B) Fluorescence spectrophotometer was used to analyse the fluorescence intensity normalised by the control group. (C) Graph displaying the increase of relative folds of ROS production normalised by the control group. Data were obtained from five independent experiments and expressed as mean ± SD. ^*^*P* < 0.01, ^#^*P* < 0.05, compared with the corresponding control or treatment group.

Supplementary Figure 3. ADM inhibits the expression of caspase-3 and caspase-7 in LPS-exposed Leydig cells. Semi-quantitative real-time PCR was performed to detect the gene expression of caspase-3 (A) and caspase-7 (D) normalised by the control group. Western blot was used to evaluate the protein level of caspase-3 (B) and caspase-7 (E). β-actin was used as internal control. Histogram displaying the densitometric analysis results of caspase-3 (C) and caspase-7 (F) normalised by the control group. The normalised levels of gene expression are expressed as ratios of the copy number of mRNA and that of β-actin cDNA. Data were obtained from five independent experiments and expressed as mean ± SD. *^*^P* < 0.01, *^#^P* < 0.05, compared with the corresponding control or treatment group.
