# Supplementary figures and images for: Adrenomedullin alleviates the pyroptosis of Leydig cells by promoting autophagy via the ROS–AMPK–mTOR axis
Source: Cell Death Dis. 2019 Jun 20;10(7):489. doi: 10.1038/s41419-019-1728-5 (PMC6586845; doi:10.1038/s41419-019-1728-5)

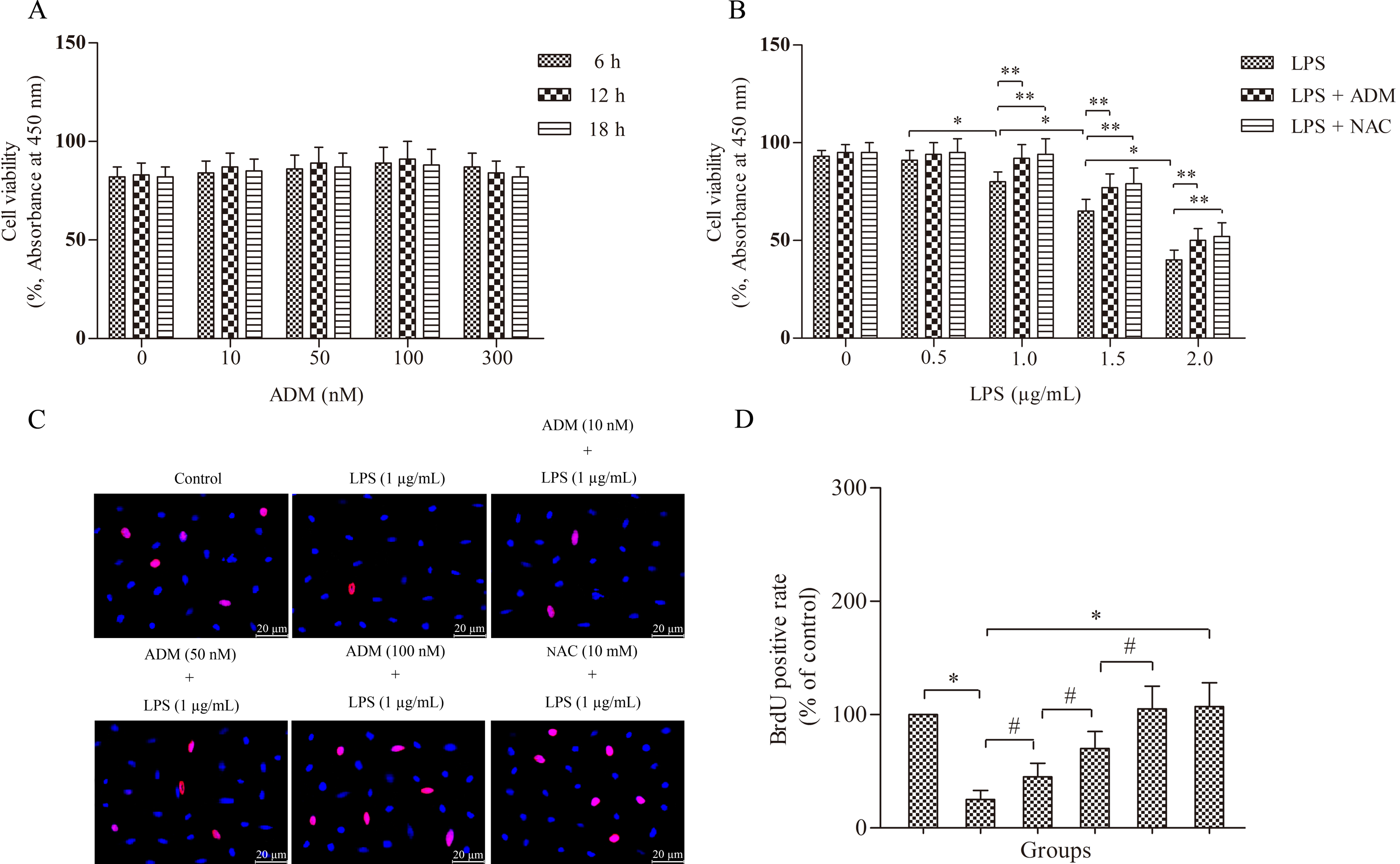

Supplement: Supplementary file 2 — Supplementary Figure 1 [file 41419_2019_1728_MOESM2_ESM.tif]

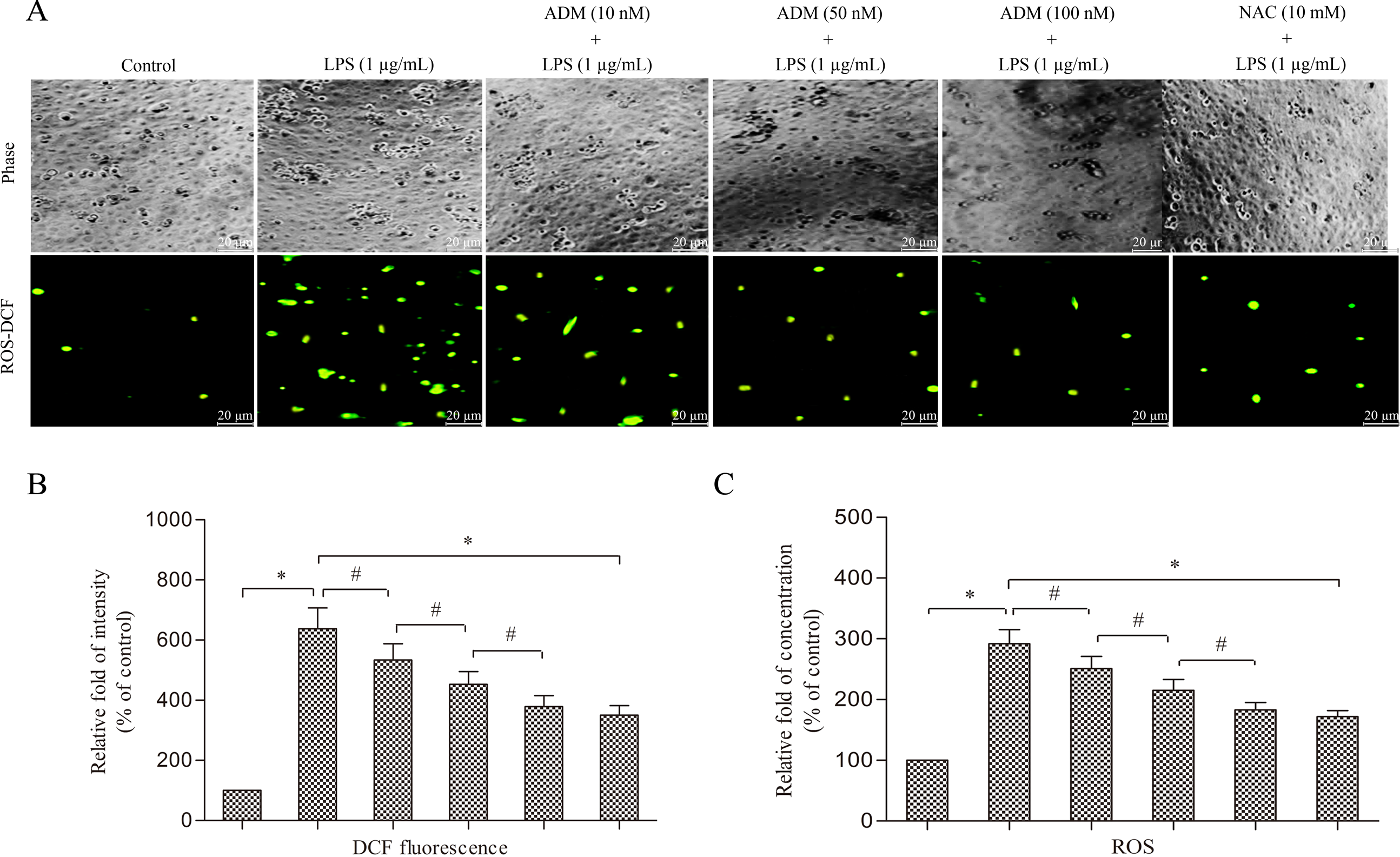

Supplement: Supplementary file 3 — Supplementary Figure 2 [file 41419_2019_1728_MOESM3_ESM.tif]

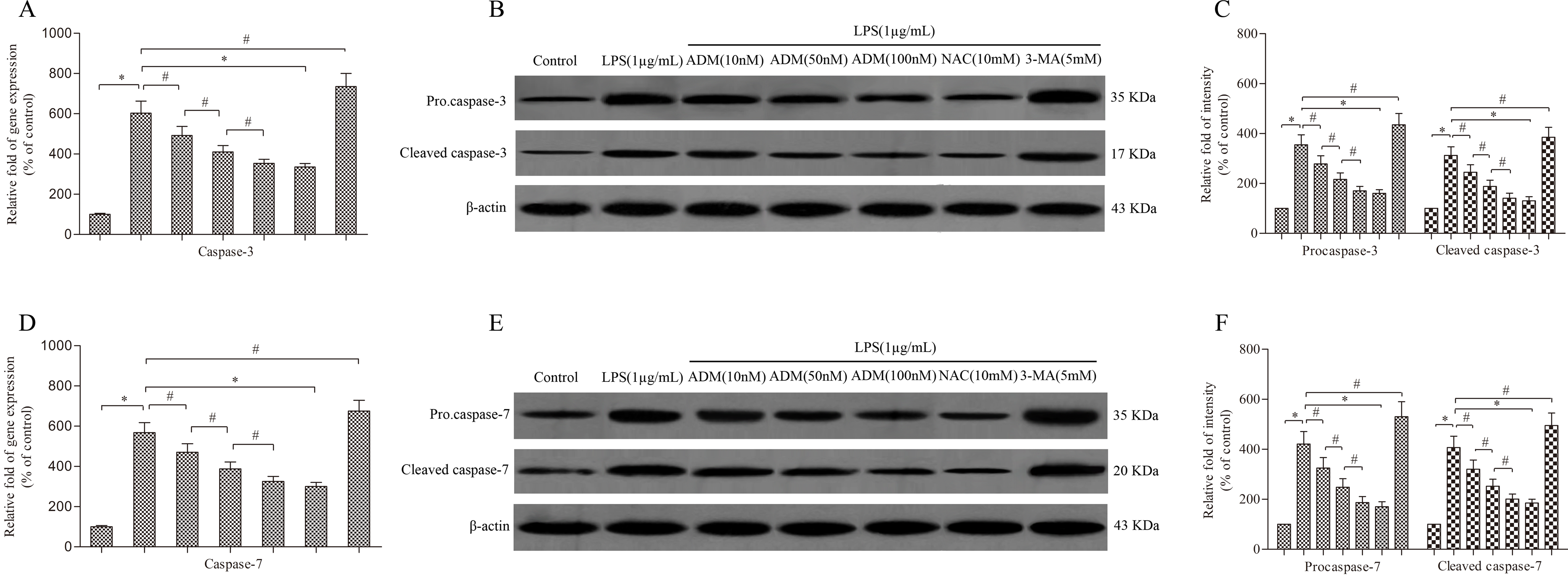

Supplement: Supplementary file 4 — Supplementary Figure 3 [file 41419_2019_1728_MOESM4_ESM.tif]
